# Finite Element Simulation and Experimental Assessment of Laser Cutting Unidirectional CFRP at Cutting Angles of 45° and 90°

**DOI:** 10.3390/polym15183851

**Published:** 2023-09-21

**Authors:** Jan Keuntje, Selim Mrzljak, Lars Gerdes, Verena Wippo, Stefan Kaierle, Frank Walther, Peter Jaeschke

**Affiliations:** 1 Laser Zentrum Hannover e.V., 30419 Hannover, Germany; v.wippo@lzh.de (V.W.); s.kaierle@lzh.de (S.K.); p.jaeschke@lzh.de (P.J.); 2 Chair of Materials Test Engineering (WPT), TU Dortmund University, 44227 Dortmund, Germany; selim.mrzljak@tu-dortmund.de (S.M.); lars.gerdes@tu-dortmund.de (L.G.); frank.walther@tu-dortmund.de (F.W.); 3 Institute of Transport and Automation Technology, Leibniz University Hannover, 30167 Garbsen, Germany

**Keywords:** laser cutting, carbon fibre-reinforced plastics, macroscopic simulation, finite element model, heat affected zone

## Abstract

Laser cutting of carbon fibre-reinforced plastics (CFRP) is a promising alternative to traditional manufacturing methods due to its non-contact nature and high automation potential. To establish the process for an industrial application, it is necessary to predict the temperature fields arising as a result of the laser energy input. Elevated temperatures during the cutting process can lead to damage in the composite’s matrix material, resulting in local changes in the structural properties and reduced material strength. To address this, a three-dimensional finite element model is developed to predict the temporal and spatial temperature evolution during laser cutting. Experimental values are compared with simulated temperatures, and the cutting kerf geometry is examined. Experiments are conducted at 45° and 90° cutting angles relative to the main fibre orientation using a 1.1 mm thick epoxy-based laminate. The simulation accurately captures the overall temperature field expansion caused by multiple laser beam passes over the workpiece. The influence of fibre orientation is evident, with deviations in specific temperature data indicating differences between the estimated and real material properties. The model tends to overestimate the ablation rate in the kerf geometry, attributed to mesh resolution limitations. Within the parameters investigated, hardly any expansion of a heat affected zone (HAZ) is visible, which is confirmed by the simulation results.

## 1. Introduction

Within the transportation sector, lightweight solutions play a vital role. The use of CFRP has gained prominence in recent years and the demand for CFRP is expected to increase further [1]. However, the increased use of these materials requires efficient processing technologies for economical, flexible, and automated production on a large scale. Conventional machining faces limitations, mainly due to significant tool wear in mechanical machining or extensive water treatment in the water-jet machining of CFRP. In addition, both mechanical and water-jet cutting apply forces to the work piece and can cause delamination, making it difficult to machine thin CFRP parts [2]. In comparison, laser-based machining has emerged as a promising alternative, offering flexibility in use and operation without contact, making it practically wear-free [3]. Nevertheless, certain challenges need to be addressed for industrial applications.

As this is a thermal-acting process, high temperatures can occur during laser material processing. While necessary for material decomposition, the generated heat can also cause degradation around the cut faces. As a result of the decomposition temperature of the matrix, which is significantly lower than for the fibres, local damage can occur within the matrix. The damage within this HAZ can manifest as delamination, cracking, and carburization [4]. The heat influence is amplified by the high thermal conductivity of carbon fibres, depending on the fibre orientation.

The relationship between the resulting temperature field, the HAZ formed due to laser processing, and selected resulting mechanical properties have been demonstrated through standardized investigations [5,6]. However, at present, it is not feasible to pre-determine these impacts based on the laser and material. To facilitate this, and to reduce the time- and resource-intensive pre-testing, it is advisable to develop a model to predict HAZ formation and adjust the process strategy accordingly.

For this purpose, modelling approaches can be utilized. Theoretical models within this context are usually based on heat flow theory. The model proposed by Kononenko [7] analytically describes heat accumulation during the pulsed laser cutting of CFRP. Kedir [8] used a similar heat accumulation theory to predict surface damage resulting from the cutting process. In addition to analytical methods, numerical simulations, such as the finite element method (FEM), enable the modelling of the process. The simulation of the laser cutting process is often related to metallic alloys [9]. Regarding composites, it is common to use three-dimensional simulation models due to the anisotropic material behaviour. Studies that deal with CFRP or different composite materials can be divided into two groups: those with a microscopic and those with a macroscopic view. Microscopic models commonly use a heterogeneous approach, separating fibre and resin, while the model size is in the magnitude of hundreds of micrometres. This approach was initially explored by Negarestani [10], who successfully simulated the fibre pull-out during laser cutting using FEM. Sun [11] applied a similar approach to model a water-jet-guided laser cutting process, while Yang [12] employed this technique to simulate a paint-stripping process for CFRP. Ohkubo [13] also employed an approach that separates the different materials within a finite volume model to investigate the thermal interaction between the hot vapor plume generated during laser processing and CFRP. These types of analyses are well suited for focussing on the ablation mechanisms and the formation of HAZ itself. However, for a process design aimed at large component sizes for industrial applications, the observation of the overall temperature evolution during the process is of crucial interest. Therefore, macroscopic simulation models that use a homogenised material model are better suited. Homogenisation is a common strategy for modelling composites, not only for laser machining. This kind of model enables the representation of direction-dependent material behaviour in a higher order of magnitude, although the detailed distinction between the fibre and matrix is omitted. For instance, Long [14] conducted a study simulating the laser ablation of CFRP using a continuous wave (CW) laser, while Moghadasi [15] employed a combination of both approaches to investigate a Kevlar/CFRP composite. While the properties of CFRP are homogenised, a weave of both materials is modelled as heterogeneous. Xu [16] employed a homogenized approach using the finite difference method to examine the ablation mechanisms of CFRP with individual and two consecutive laser pulses, taking phase transitions into consideration. In this study, a macroscopic simulation model will be used, because the interest of the study lies in the global behaviour, which is essential for an industry-oriented target setting.

## 2. Materials and Methods

### 2.1. Experimental

**Figure 1:**
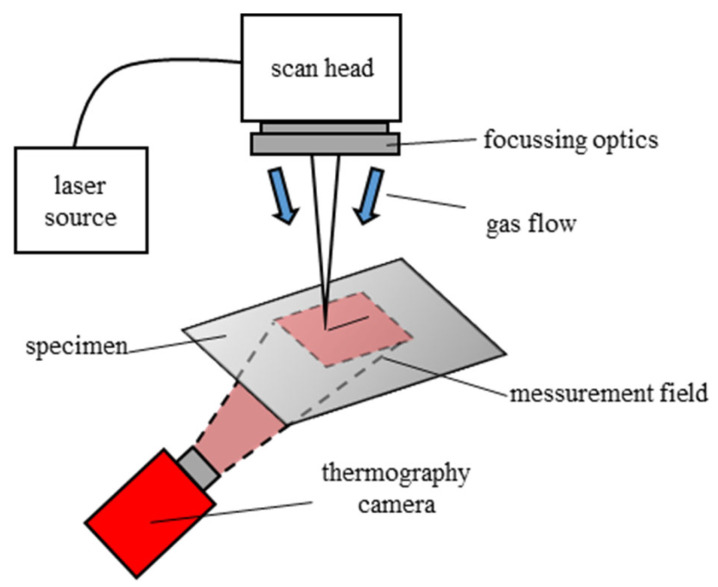
Schematic view of the experimental setup.

A thin-disk high-power nanosecond pulsed laser, the TruMicro 7050 from TRUMPF Laser GmbH, Ditzingen, Germany, with a maximum output power of 1.5 kW, was used for the cutting experiments. The laser emitted at a wavelength of 1030 nm. The laser radiation was guided by an optical fibre to a scanning optic (scan head). The focal point had a tophat intensity profile with a spot diameter of 1.2 mm. The scan head guided the laser beam over the material surface of the CFRP. The pulse duration was 30 ns, with a frequency ranging between 5 and 50 kHz. This setup demonstrated its suitability for CFRP processing, as it enabled rapid processing speeds while maintaining a high cutting quality [17]. A gas flow was applied using a ring jet to protect the focusing optics from ablation products. This caused a cooling effect on the material surface, which needed to be considered in the simulation model. The experimental setup is schematically shown in Figure 1.

A repetition rate of 18.8 kHz was specified because, at this rate, a maximum average output power of 1.5 kW and a maximum pulse energy of 80 mJ were available. For the laser cutting of CFRP, it is common to use a multipass cutting strategy where a cut-through is achieved by applying several laser beam passes over the workpiece. A single pass is also referred to as a scan. This strategy is the basis for laser cutting and was used to achieve small or no HAZ. After each laser pass, the process heat was conducted into the material, and the material directly at the cutting-edge cooled down. This effect was further enhanced by implementing a break in between each laser pass. As a high number of scans might be necessary to achieve a cut though based on the selected laser parameters, the evaluation of this model so far was based on 5 scans. This not only reduced the computation time and enabled the early assessment of the model, but also allowed for an examination of the heat accumulation beneath the cutting area.

The cutting length was set to 20 mm, the velocity to 1000 mm/s, and the pause time in between the passes was set to 600 ms. To investigate the effect of fibre orientation, cuts were performed at 45° and 90° cutting angles ($\alpha_{cut})$, defined relative to the uniform fibre direction of the material. The comparison between the experiment and simulation was performed through an evaluation of the surface temperatures and using cross sections made from the cut specimens, enabling the detection of the material ablation depth and possible HAZ formation.

The laser cutting process was captured using a thermographic camera. The thermography system was placed below the specimen to observe the bottom surface temperature. This setup ensured that recordings were shielded from process emissions, ablation products, or reflections that may have influenced the measurements. Additionally, by using the measured temperature of the material on this side, the thermal conductivity properties used in the simulation model could be evaluated. For this purpose, the ImageIR^®^ 8300 thermographic camera system from Infratec GmbH (Dresden, Germany) was utilised. The camera was placed at a distance of 0.5 m and an angle of 45° relative to the bottom surface of the specimen. The selected settings allowed for a measurement frequency of 250 Hz at a quarter-frame window. This provided a measurement field of 38 × 48 mm^2^ with a pixel size of 75 µm.

### 2.2. Finite Element Simulation Model

#### 2.2.1. Transient Thermal Analysis

In this study, a transient thermal finite element model was developed, utilizing temperature as the sole solution variable (degree of freedom). The interaction of the laser beam with the surface was implemented as a time-varying surface heat load. The material removal was achieved using the element-death method, a simplified approach where elements are deactivated from the model once they reach a certain critical temperature threshold. Previous investigations [10,11,12,14,15] demonstrated that this technique is suitable for simulating the material removal process during laser cutting. When the focus was set to a larger scale, it was acceptable that phenomena such as the phase transition, vaporization effects, and vapour or melt dynamics, which occur during the laser material interaction, were neglected. Simplification was also necessary for modelling the heat source on the surface. Although the laser operated in a pulsed mode, the heat source was estimated as an adequately adopted continuous working laser source. The pure number of pulses (working with a frequency in the kilohertz range) to be simulated was already substantial. For instance, using a cutting length of 20 mm, a velocity of 1000 mm/s, and a frequency of 18.8 kHz would yield 376 pulses. Additionally, a more microscopic view of the process is required when examining the material interaction at the level of individual laser pulses. This perspective would also bring the ablation mechanism back into relevance. Both factors would lead to an extensive computation time and were impractical for this macroscopic investigation. Therefore, to represent the energy input by the pulsed laser, the absorbed laser power $P_{in}$ per scan was calculated, taking into account the number of pulses $N_{P}$, the pulse energy $E_{P}$, and the cutting time per scan $t_{cut}$, yielding the overall output power P of the laser. An absorption coefficient ($\eta_{abs})$ of 0.9, as stated by Freitag [18], was also considered:

(1) $$t_{cut}=\frac{L}{v_{cut}}$$

(2) $$N_{P}=t_{cut}\times f$$

(3) $$P_{in}=\frac{N_{P}\times E_{P}}{t_{cut}}\times \eta_{abs}=P\times \eta_{abs}$$

**Figure 2:**
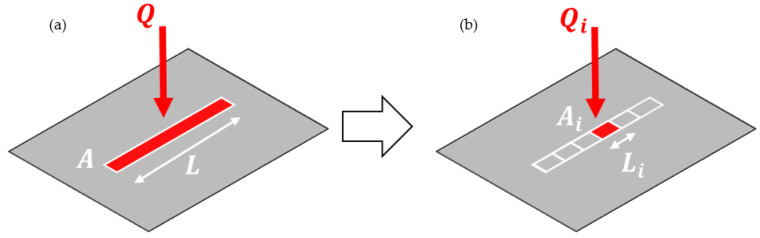
Different laser cutting simulation strategies: (**a**) summed up heat load applied at once and (**b**) adopted heat load applied step by step.

The power was then applied as a heat flux boundary condition to the cutting area on the material surface, simplified using the cutting length L and the spot diameter $d_{spot}$. In the initial approach, the entire energy was applied at once for the duration of the cutting time, without considering the forward movement characteristics of the cutting process. The temperatures obtained from this approach significantly exceeded the measured data. To address this, the cut was divided into equal time steps, and the power was applied step by step. This procedure is schematically shown in Figure 2. The number of steps and their corresponding step sizes were determined iteratively. Increasing the number of steps reduced the remaining heat as elements were deactivated after each step when they exceeded the sublimation temperature. A low number of steps resulted in longer step times and a longer period during which the heat dissipated into adjacent areas until relevant elements were deactivated. Finally, the thermal load was calculated as the heat flux per step using the following equations:

(4) $$Q_{HF,i}=\frac{P_{in}}{A_{i}}$$

(5) $$A_{i}=\frac{d_{spot}\times L}{N_{steps}}$$

In a preliminary study [19], the maximum process temperatures on the top of the surface were compared with the experimental data. Through an iterative process, it was found that using 25 divisions per cut provided a good agreement, while maintaining an acceptable calculation time. The chosen number of steps was suitable for the investigated parameters, but it may need adjustment for other parameter settings. This approach adopted a CW process based on the high frequency of 18.8 kHz, while the energy input was calculated according to the pulsed process. Between multiple laser beam scans and after the last scan, the heat flux was set to zero to simulate the cooling phases without the laser material interaction.

Alongside the heat source, two different convection boundaries were considered. The first was convection to the surrounding air, with an ambient temperature of 28 °C, according to the current room temperature and a constant convection coefficient of 10 $W/(m^{2}K)$. Another convection boundary was applied to the top surface, due to the air-flow caused by the ring jet. This coefficient was calculated based on flow against a vertical plane [20] and was set to 300 $W/(m^{2}*K)$. More assumptions that had to be accounted for were as follows:

- The bulk and surrounding temperature was set to 28 °C.
- Radiation heat loss was neglected.
- Thermophysical material properties were assumed to be constant.
- Pores or other material defects were neglected.
- The laser beam energy distribution was uniform and ideal.

During the laser−material interaction, a constant time step size was used in the simulation. To save computation time within the simulation model, the step size was increased when the laser was turned off. This adjustment did not impact the simulation accuracy as the temperature gradients in this phase were lower.

#### 2.2.2. Geometry

**Figure 3:**
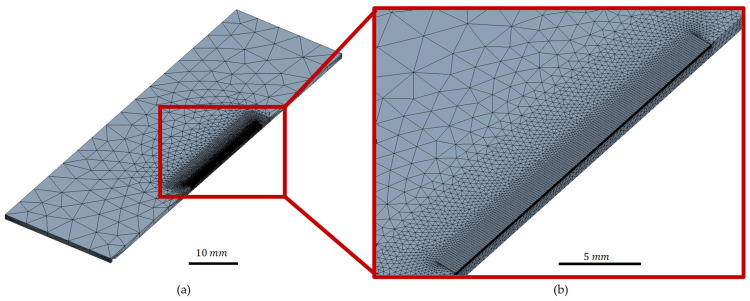
Finite element model: (**a**) larger scale and a (**b**) close-up of the cutting zone.

A three-dimensional rectangular body with dimensions of 80 × 25 × 1.1 ${mm}^{3}$ was created as the representative geometry. To reduce the simulation effort, a half-model with a symmetry plane in the middle of the cut was utilised. For the cutting zone, a structured (hexagonal) mesh with a finer resolution was employed to achieve a good accuracy, given the expected high temperature gradients during the simulation. Additionally, the coordinates of the structured mesh were known and thus controllable. The mesh size in the thickness direction was set to 10 µm within the finely resolved area. Surrounding the cutting area, a free (tetrahedral) mesh was implemented to increase the element size and reduce the number of elements in regions where only small temperature gradients were anticipated. The analysis employed eight-node hexagonal elements for the structured mesh and five-node tetrahedral elements for the free mesh. The linear element type SOLID70 was selected for all elements, as it possessed the necessary three-dimensional conduction capability for transient thermal analysis. The model, depicted in Figure 3, consisted of a total of 246,982 elements and 182,775 nodes.

#### 2.2.3. Material Properties

The investigated material was a unidirectional CFRP composed of six layers of carbon fibre tape and an epoxy resin, with a thickness of 1.1 mm. The fibre volume ratio was 59.2%. The properties were provided by the supplier, ERC GmbH, and supplemented with values from the relevant literature [18,21]. The properties of the composite laminate, including the density, thermal conductivity, and specific heat capacity, were determined according to the rules of mixture (ROM) and the classical laminate theory (CLT) [22,23]. The ROM weighed the composite properties as the average of the component properties, considering the fibre volume fraction. CLT accounted for the different material orientations in the layers. In this study, as a unidirectional composite was investigated, it was not necessary to apply CLT. The unidirectional structure of the material resulted in a significant difference in heat conductivity between the fibre direction and the perpendicular direction, which was decisive for the temperature expansion behaviour. The evaporation temperatures for the fibres and the matrix were obtained from the literature. The glass transition temperature for the epoxy resin, approximately 200 °C, was determined through differential scanning calorimetry and was considered to be the value for damage initiation. All of the properties are summarised in Table 1.

## 3. Results and Discussion

Laser cutting of unidirectional CFRP was performed. The temperature was measured with a thermography camera and predicted with the simulation model. After cutting, the kerf geometry was investigated with a laser scanning microscope (LSM), from the KEYENCE DEUTSCHLAND GmbH, Neu-Isenburg, Germany, to measure the ablation depth. Additionally, cross sections were made from the cut specimens, enabling the investigation of the kerf geometry and possible damage to compare it with the simulation.

### 3.1. Temperature

**Figure 4:**
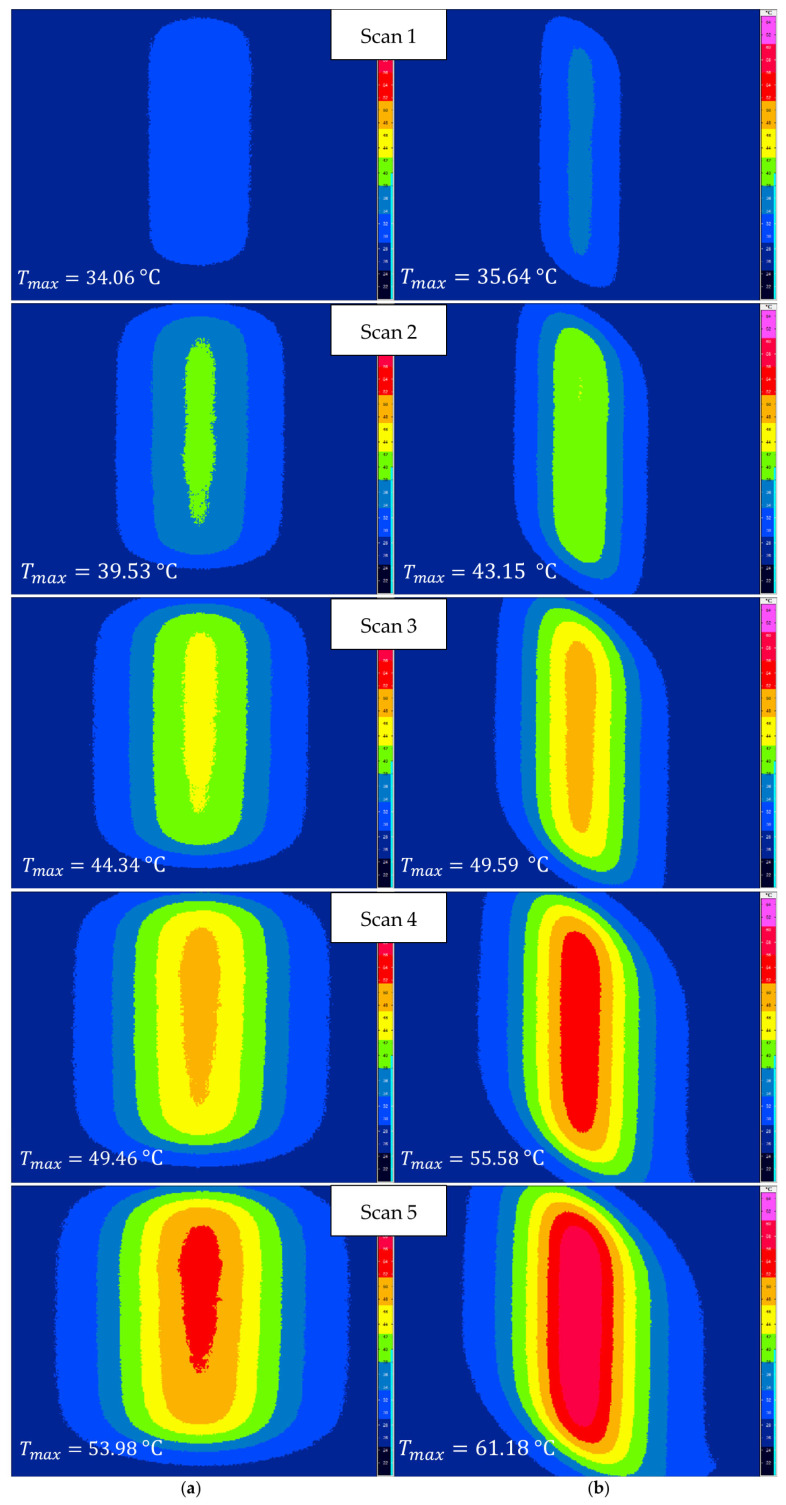
Thermographic imaging of the bottom side temperature field expansion over time: (**a**) at a 90° cutting angle and (**b**) at a 45° cutting angle.

**Figure 5:**
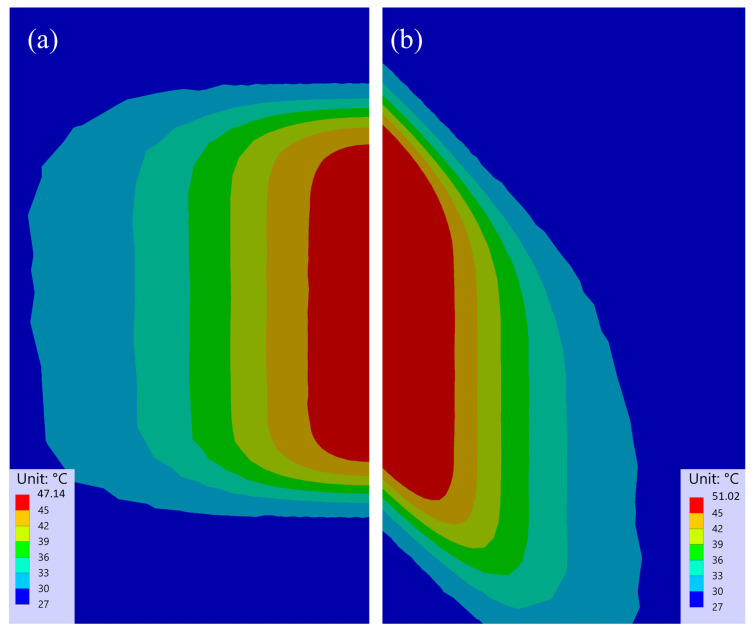
Simulated temperature fields 500 ms after the fifth laser beam scan: (**a**) at a 90° cutting angle and (**b**) at a 45° cutting angle.

The overall temperature expansions of the experiments at different cutting angles are depicted in Figure 4. The thermography images illustrated the temperature fields on the bottom side of the specimen 500 ms after each laser beam scan. All of the provided thermographic images shared the same scale. The images show the steady propagation of temperature over time. Additionally, the difference in fibre orientation was visible, and was attributed to the higher thermal conductivity in the fibre direction. Not only was the shape of the temperature distribution different, but the maximum temperatures at the 45° cutting angle were also higher compared with the 90° cutting angle. The simulated temperature field at 500 ms after the last scan for both cutting angles is presented in Figure 5, allowing a qualitative comparison. The simulation confirmed both the shape of the temperature field and the distinct temperature levels. However, due to the different scales between the simulation and the thermographic recordings, this type of temperature representation was not ideal for a direct comparison.

While the simulation model accurately described the overall behaviour, deviations arose when examining the exact temperatures. For the comparison of the simulation temperatures with the experimental temperatures, two positions at the bottom surface were used: the first ${(s}_{1})$ was directly in the centre underneath the cut and wsa thus located directly beneath the impact area of the laser. The other position $\left(s_{2}\right)$ was placed 6 mm horizontally away from the first position so as to gain knowledge about the temperature flow into the material.

Thermographic imaging involves some uncertainties that needed to be considered. An ideal setup would require a measurement angle of 90° relative to the specimen, but technical limitations restricted the angle to 45°. Another source of variance was the emission coefficient of the specimen. As the CFRP was black, the emission coefficient was expected to be close to one, while in reality, it is very likely to be lower [24]. These uncertainties were addressed by considering a delta of ∆T=±2°C around the measured temperature according to Bluemel [25]. Additionally, the real surrounding temperature was not perfectly constant as assumed in the simulation. To facilitate a better comparison with the simulation values, the measured temperature at any time $T_{i}$ was normalised to an initial temperature of 28 °C:

(6) $${∆T}_{norm}=28{}^{\circ}C-T_{0}$$

(7) $$T_{i,norm}=T_{i}-{∆T}_{norm}$$

To take measurement errors into account, the measured temperature was shown using two boundary curves that enclosed the potential range of fluctuation. Additionally, slight variations in spatial resolution needed to be considered as the temperature data were provided for a specific pixel size and position, leading to a maximum possible position error equal to half the pixel size. Similarly, the simulated temperatures were associated with specific node locations. If a node and a pixel position did not align, linear interpolation was employed between the temperatures of the nearest nodes.

**Figure 6:**
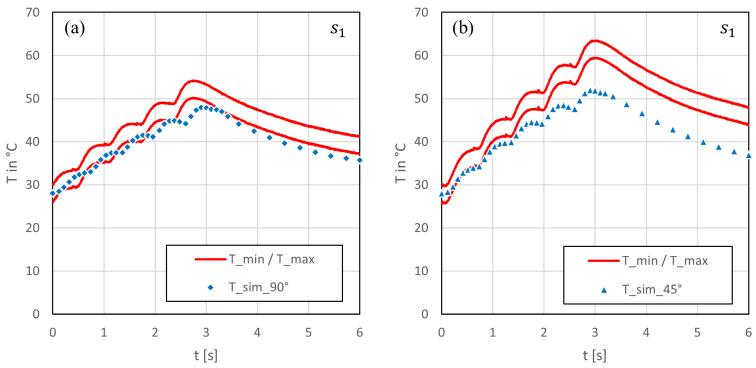
Comparison of the simulated and measured bottom surface temperature history in the centre of the kerf: (**a**) at a 90° cutting angle and (**b**) at a 45° cutting angle.

**Figure 7:**
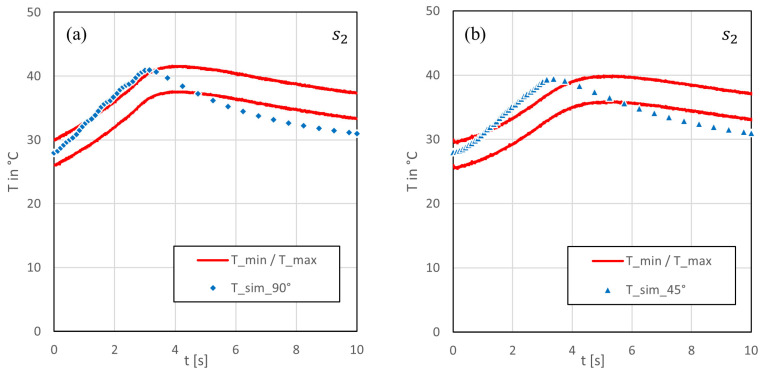
Comparison of the simulated and measured bottom surface temperature history at a 6 mm distance to the kerf: (**a**) at a 90° cutting angle and (**b**) at a 45° cutting angle.

In general, the temperature plots shown in Figure 6 and Figure 7 can be divided into two phases: the heating phase and the cooling phase. The heat accumulation caused by five laser beam scans was visible in the experimental and simulation results at the centre of the laser beam cut ($s_{1}$), as shown in Figure 6. This affect was independent of the cutting angle. At a 90° cutting angle (Figure 6a), the simulation results aligned closely with the lower boundary of the measured temperature variation range. However, a small delay in temperature rise was observed between the experiment and simulation, resulting in a local shift in maxima. The difference between the maximum temperatures between the experiment and simulation was approximately 4 °C, and the simulation reached this temperature 250 ms later. The slower temperature rise indicates that the conductivity in the through-thickness direction used in the model was too low. At a 45° cutting angle, Figure 6b, the difference between the measured and simulated data increased to a maximum of nearly 7 °C. As observed earlier, the centre became hotter at a 45° angle compared with a 90° due to the lower lateral dissipation of heat along the fibres. Given that the rise in simulated temperature was notably lower than in the experiment, it is plausible that not only did deviations exist in the thermal conductivity, but that the overall heat input, which lead to the temperature rise, might have also been too low within the model.

Comparing the cooling phases, it can be observed that the curves exhibited a similar slope once they reached their respective maximum temperatures. However, during temperature decay, there was a nearly constant offset between the simulated and measured data, depending on the difference in maximum temperature and the time offset.

At a greater distance from the center of the cutting kerf ($s_{2}$), see Figure 7a,b, the disparity between the two cutting angles became notably smaller, for both the experiment and simulation. The detected temperatures in point $s_{2}$ showed a steadier rise than directly beneath the cut $\left(s_{1}\right)$. In the simulation, the maximum temperatures reached approximately 42 °C at a 90° and a 40 °C at 45° cutting angle. Both values were close to the defined upper boundary of the experimental data. Additionally, within the simulation, the temperature peak was reached about 1 s earlier at 90° and around 2 s earlier at 45° in contrast with the experiment. Furthermore, in comparison, the simulated temperatures exhibited a steeper ascent and a faster descent after reaching the maximum at both cutting angles, compared with the temperatures measured for the experiment. These observations imply that heat flowed too fast in lateral direction within the model. From this, it can be deduced that the chosen thermal conductivity in the fiber direction might have been be too high.

Overall, the model could capture the heat conduction behavior in principle, but there were noticeable deviations that may have been attributed to the imprecise ratio of the thermal conductivity in the three spatial directions, which were used in the simulation. Another reason for the deviations in the temperature profiles could be as a result of the material removal. If the simulation removed more material than the actual experiment, additional energy was required for this process, which consequently was not available for heating through conduction. To investigate this further, a comparison of the ablated materials was conducted.

### 3.2. Ablation Depth

The ablated depth was determined with the aid of LSM. The ablation depth $d_{abl}$ was measured using cut specimens from one and five laser beam scans. Additionally, the rate of ablation $∆d_{abl}$ per scan was calculated based on this data. For a single scan, these values were identical and for five scans, they were calculated as follows:

(8) $$∆d_{abl,5}=\frac{d_{abl,5}-d_{abl,1}}{5-1}$$

The results are summarised in Table 2. After a single laser beam scan, the experimental ablation depth at a cutting angle of 45° measured 44.98 µm, slightly less than the measurement of 54.76 µm at 90°. However, considering the standard deviation, the maximum difference between them was merely 5.02 µm.

**Figure 8:**
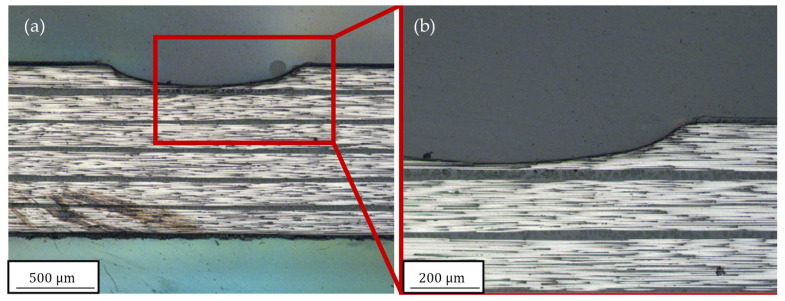
(**a**) Cross sections after five scans at a 90° cutting angle and (**b**) close-up.

**Figure 9:**
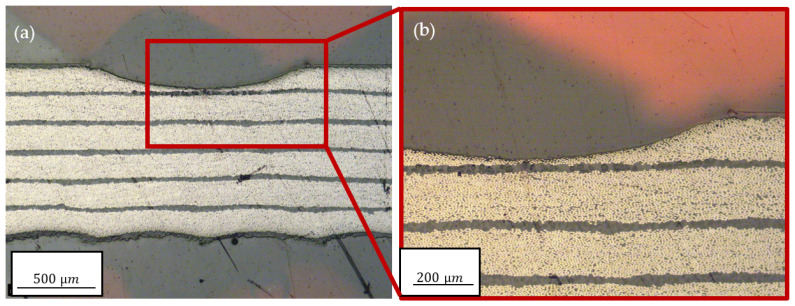
(**a**) Cross sections after five scans at a 45° cutting angle and (**b**) close-up.

When comparing the experimental ablation rates after one and five scans, it is clear that the ablation rate was higher for the first scan, and was unaffected by the angle. This can be attributed to a change in geometry from a straight surface to a spherical kerf and a change in surface roughness, which modified the absorption behavior for the laser radiation. In the cross section shown in Figure 8 and Figure 9, it can be observed that there was an epoxy layer on top of the laminate, which was easier to remove compared with the fiber−matrix compound. This is not considered in the model.

The simulation also exhibited a slightly higher ablation depth at a 90° angle than at a 45° angle after five scans, corresponding to the experimental results. When examining the ablation rate, it is evident that the model assumed a more constant rate, whereas in the experiment, the rate changed. It is conclusive that the difference in ablated volume and, thus, the removed heat energy influenced the temperature field expansion, as previously inferred.

**Figure 10:**
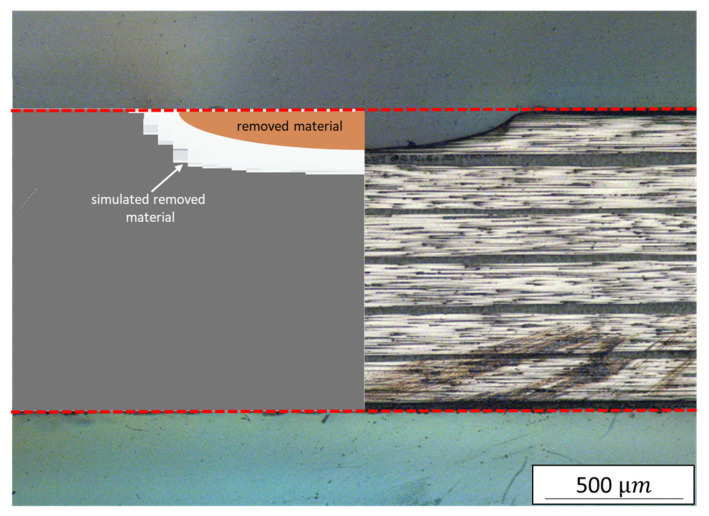
Comparison between the simulated and real kerf geometry after five scans at a 90° cutting angle; red dashed lines indicate the original shape of the sample.

To further investigate the differences in ablation behaviour, the kerf geometries were compared, as shown in Figure 10. The simulation model accurately represented the spherical ablation shape to the extent permitted by the mesh resolution. However, the model overestimated the overall ablated volume after five scans. The reasons for these deviations have not yet been conclusively clarified. Possible factors could include differences in material properties, such as the assumed sublimation temperature of 3650 °C. Another factor could be the method employed for element deactivation. Only complete elements can be deactivated, so if a single node of an element reached the sublimation temperature, the entire element was deactivated, resulting in more material being removed than necessary. The greatest impact likely stemmed from the amount of heat input, which depended on the geometry of the kerf and how it was transferred to the model.

### 3.3. Heat Affected Zone

The selected parameters used in this study did not result in significant HAZ. To generate more heat input during the cutting process and potentially observe a larger HAZ, additional scans and different laser settings, such as a lower scanning velocity, would be required. However, the simulation results confirm that no HAZ was present when examining the maximum temperatures around the kerf. The remaining temperature reached its highest value immediately after the material removal step in the simulation. It should be noted that the maximum temperature depended on the simulation time step size immediately after the ablation. With smaller time steps, the remaining temperature after material removal would be higher. If the time step was small enough, this would consistently result in temperatures close to the deactivation temperature of the elements, which would exceed the damage initiation temperature of the epoxy resin.

**Figure 11:**
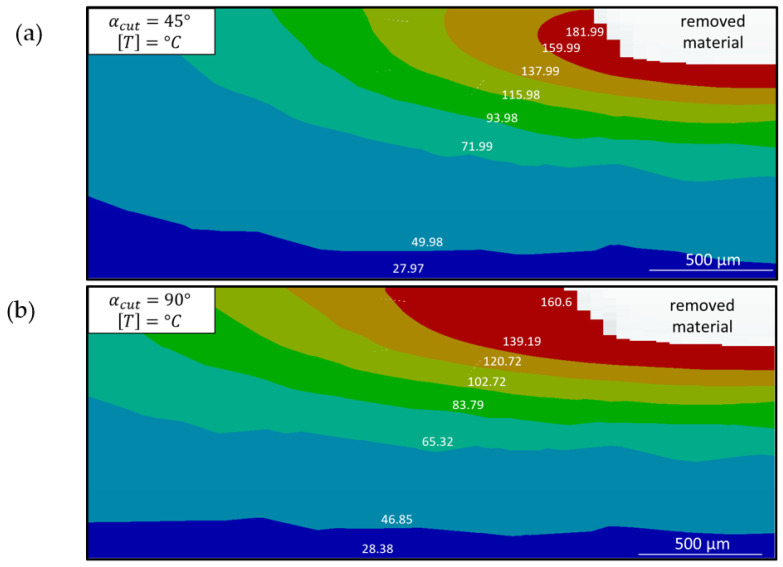
Simulated temperature plot on the cross section in the centre of the cut: (**a**) at a 45° cutting angle and (**b**) at a 90° cutting angle.

Using a time step of 100 ms, the temperature distributions shown in Figure 11 were obtained. The maximum temperatures remaining were 160.6 °C and 181.99 °C, respectively. These temperatures were lower than the damage initiation temperature and, thus, consistent with the experimental observation, as seen in Figure 8b and Figure 9b. Apart from minimal dark discolouration, which was also observed on all surfaces of the sample, no quantifiable expansion of HAZ could be detected. Another noteworthy finding is that no temperature exceeding 200 °C persisted for longer than 100 ms. This suggests that the damage temperature needed to be surpassed for a longer duration to result in visible damage.

## 4. Conclusions

This study investigated the laser cutting process of unidirectional CFRP at different cutting angles through a combination of experiments and numerical simulations. The results provided insights into the temperature expansion, ablated material, and occurrence of HAZ. The measurement of the bottom surface temperature allowed for consideration of the conductive behaviour of the material in both the thickness and crosswise directions during cutting. In general, the simulation model was able to predict the effects of different cutting angles on the temperature field, such as the overall shape and higher temperature levels at a 45° angle. This indicates that the homogenised material model was suitable for representing the global behaviour of the material.

Beneath the centre of the laser cut, the model exhibited a gradual increase in temperature, as observed in the experiment, albeit with a difference in maximum temperature of approximately 4 °C less at a 90° cutting angle and around 7 °C at a 45° cutting angle. Further away from the cut, the temperature history in the model followed a steadier rise, similar to the experiment. The maximum temperatures aligned at 42 °C (90°) and 40 °C (45°). However, distinctions in the slope between the temperature ascent and descent were evident. Suspected reasons for these variations include the assumed material properties, particularly the ratio of thermal conductivity in the fibre direction versus across it. The assumed radial conductivity of the fibre may have been too low. Another factor contributing to the deviations was the difference in ablated material volume, affecting the remaining heat energy dissipation into the material. It is shown that the model assumed a linear removal rate per laser beam scan, whereas the experimental results indicate a change in the removal rate, especially after the first pass. This caused an overestimation of the ablation depth with maximum differences of 47.53 µm (45°) and 52,4 µm (90°). The reasons behind these deviations will be further investigated in future research, exploring factors such as the sublimation temperature, reflectivity, and absorptivity of the material, respectively, as well as the surface topology.

Regarding the damage, no visible HAZ could be identified in the cross section at the employed parameters. An analysis in this regard is consequently limited. However, the temperatures within the model also remained below levels that would suggest the presence of damage.

In terms of improving the accuracy of the model, it would be advisable to adjust the element size precisely on the edge of the laser spot, particularly when considering the shape of the kerf geometry. Additionally, to evaluate the model’s predictive capability and investigate the effect of the HAZ on material strength, it is important to intentionally create a measurable extension of the HAZ by adapting the experimental parameters.

Furthermore, in future investigations, it will be explored whether any effects resulting from heat input during laser cutting have occurred, despite the absence of a visible HAZ. To achieve this, it would be appropriate to conduct coupled thermal and stress analyses to gain insight about the thermal and structural interaction.

## Figures and Tables

**Table 1:** Simulation material properties: from the ^(a)^ supplier and from the ^(b)^ literature [18,21].

Material Property	Carbon Fibre	Epoxy Resin	Laminate
Density, $\rho (kg/m^{3})$	1780 ^(a)^	1280 ^(a)^	1575
Specific heat capacity, c Jkg·K	875 ^(a)^	1200 ^(a)^	982.7
Thermal conductivity, k Wm·K	║50 ^(b)^ ┴ 5 ^(b)^	0.2 ^(a)^	║31.64 ┴ 0.68
Thermal diffusivity, α m2s	║${3.21\times 10}^{-5}$ ┴ ${3.21\times 10}^{-6}$	$1.3\;{\times 10}^{-7}$	║${2.04\times 10}^{-5}$ ┴ $4.39{\times 10}^{-7}$
Evaporation temperature, $T_{s}$ $\left({}^{\circ}C\right)$	3650 ^(b)^	500 ^(b)^	−

**Table 2:** Ablation depth and rate measured in the simulation.

	No.	$\alpha_{cut}$	$N_{rep}$	$d_{abl}\;in\;\mu m$	$∆d_{abl}\;in\;\mu m$
Experiment	E1	45	1	44.98±3.17	44.98
	E2	45	5	151.26±4.71	26.57
	E3	90	1	54.76±1.59	54.76
	E4	90	5	159.99±4.92	26.30
Simulation	S1	45	1	43.38	43.38
	S2	45	5	198.79	38.14
	S3	90	1	43.83	43.83
	S5	90	5	212.39	41.87

## Data Availability

The data used to support this study’s findings can be provided by the corresponding author upon reasonable request.
